# A Farnesyltransferase Acts to Inhibit Ectopic Neurite Formation in *C*. *elegans*

**DOI:** 10.1371/journal.pone.0157537

**Published:** 2016-06-14

**Authors:** David Carr, Leticia Sanchez-Alvarez, Janice H. Imai, Cristina Slatculescu, Nathaniel Noblett, Lei Mao, Lorena Beese, Antonio Colavita

**Affiliations:** 1 Neuroscience Program, Ottawa Hospital Research Institute, Ottawa, Ontario, Canada; 2 Department of Cellular and Molecular Medicine, University of Ottawa, Ottawa, Ontario, Canada; 3 Department of Biochemistry, Duke University Medical Center, Durham, North Carolina, United States of America; 4 University of Ottawa Brain and Mind Research Institute, Ottawa, Ontario, Canada; McGill University, CANADA

## Abstract

Genetic pathways that regulate nascent neurite formation play a critical role in neuronal morphogenesis. The core planar cell polarity components VANG-1/Van Gogh and PRKL-1/Prickle are involved in blocking inappropriate neurite formation in a subset of motor neurons in *C*. *elegans*. A genetic screen for mutants that display supernumerary neurites was performed to identify additional factors involved in this process. This screen identified mutations in *fntb-1*, the β subunit of farnesyltransferase. We show that *fntb*-1 is expressed in neurons and acts cell-autonomously to regulate neurite formation. Prickle proteins are known to be post-translationally modified by farnesylation at their C-terminal CAAX motifs. We show that PRKL-1 can be recruited to the plasma membrane in both a CAAX-dependent and CAAX-independent manner but that PRKL-1 can only inhibit neurite formation in a CAAX-dependent manner.

## Introduction

Initial neurite formation and extension is a key stage in the transition from neuroblast to mature neuron. Cell intrinsic factors that orchestrate the localized regulation of actin and microtubules determine the site of nascent neurite outgrowth [1–4]. In the complex environments existing in *vivo*, these factors are polarized at sites of neurite outgrowth in response to extracellular cues [5,6]. However, in addition to neurite promoting factors, mechanisms that inhibit neurite formation help to sculpt neuronal connections by blocking inappropriate neurites during development and by maintaining neuronal morphology as neurons age [7,8]. The genetic pathways involved in inhibiting neurite formation remain poorly understood.

The Frizzled/Planar Cell Polarity (Fz/PCP) pathway, a non-canonical Wnt pathway, is involved in mediating polarized cytoskeletal rearrangements that underlie several developmental processes including ordered cell movements during gastrulation, neuronal migration and axon guidance [9,10]. In *C*. *elegans*, a PCP-like pathway that includes the core PCP proteins PRKL-1/*Prickle* and VANG-1/*Van Gogh* acts in a subset of motor neurons to block neurite formation along the anterior-posterior (AP) axis in order to restrict neurite outgrowth to an orthologous axis [11]. PRKL-1-mediated neurite inhibition, at least in part, appears to involve localization to the plasma membrane of neuronal somas. However, other components of this pathway, in particular those which act in membrane targeting remain to be discovered.

Herein, we present the findings of a genetic screen for mutations that, like those in *prkl-1*, fail to block inappropriate neurite formation from neuronal somas. In addition to new *prkl-1* and *vang-1* alleles, this screen identified mutations in *fntb-1*, the β subunit of farnesyltransferase (FTase). FTases are cytoplasmic proteins that transfer a prenyl (farnesyl) group to C-terminal CAAX-motif containing proteins [12]. Prenylation is an important post-translational modification that mediates protein insertion into cellular membranes. We show that FNTB-1 acts cell autonomously in motor neurons and that the *fntb-1* mutations recovered in our screen are predicted to disrupt FTase activity. CAAX-containing Prickle proteins are well established as evolutionary conserved targets of FTases [13,14]. We show that the PRKL-1 CAAX motif is required to block ectopic neurite formation and that PRKL-1 is targeted to the plasma membrane of neuronal somas in both a CAAX-dependent and CAAX-independent VANG-1-dependent manner.

## Materials and Methods

### Genetics

Worms were maintained at 20°C on *E*. *coli*-seeded nematode growth medium plates. The N2 wild-type strain and the following alleles obtained from external sources were used in this study: LGX: *vang-1(tm1422)*, LGIV: *prkl-1(ok3182)*, *fnta-1(ok269)*. LGV: *fntb-1(ok590)*. VC4 and VC5 neurons were visualized using *cyIs4[Pcat-1*::*GFP pRF4(rol-6)](V)*.

### Genetic screen for VC4 and VC5 neurite defects

Worms carrying the VC4 and VC5 reporter transgene *cyIs4[Pcat-1*::*GFP pRF4(rol-6)]* [15] were mutagenized with 50mM ethylmethanesulfonate (EMS) as described by Brenner [16]. Young adult F1 progeny of these worms were then transferred to freshly seeded plates (5 F1 worms/plate) and allowed to self-propagate. 30–40 young adult roller progeny from each F1 plate were then transferred in a drop of M9 to slides prepared with 2% agarose pads and covered with glass cover slips. These worms were visually screened for AP-directed ectopic VC4 and VC5 neurites under 20x magnification on an AxioplanII fluorescence microscope. The roller background facilitated the visualization of VC4 and VC5 neurites on the ventral side and ensured that worms, embedded in the agarose pad, were immobilized. Worms displaying a VC neurite defect were recovered from the slide by carefully sliding off the cover slip and transferring individual worms to fresh plates to self-propagate. Potential mutants were rescreened to authenticate the outgrowth defect and then outcrossed at least twice before further characterization.

### GFP reporters, rescue constructs and transgenic strains

An *fntb-1* cDNA minus its stop codon was amplified by RT-PCR from a mixed-stage N2 RNA preparation and cloned upstream and in frame with the GFP cassette in pPD95.77 (pAC248). This plasmid was used to generate *fntb-1p*::*FNTB-1*::*GFP* by inserting 485 bp of *fntb-1* promoter sequence amplified from N2 genomic DNA. The transcriptional *fntb-1p*::*GFP* reporter was made using the same promoter sequence inserted into the polylinker site of pPD95.77. *unc-4p*::*FNTB-1*::*GFP* was generated by stitching together two PCR products using an overlap extension PCR approach. The *unc-4* promoter fragment was amplified from *unc-4p*::*GFP*::*PRKL-1* (pAC100) [11] and the *fntb-1* cDNA and *unc-54* 3’UTR fragment from pAC248. The CAAX-deleted PRKL-1 plasmid *unc-4p*::*PRKL-1*::*ΔCTVS* was made by replacing the full-length *prkl-1* cDNA in pAC100 with a PCR amplified cDNA that lacked the C-terminal CTVS codons. Gibson assembly using pAC100 as a template was used to make the *unc-4p*::*PRKL-1*::*CNIM* construct in which C-terminal *prkl-1* residues (VRMAKKKKSSRCTVS) were replaced with the C-terminal residues of *mig-2* (LHPKPQKKKKsssSCNIM). All cloning primers are listed in S1 Table. Transgenic strains were generated by injecting constructs at 40 ng μl^-1^ with either 40 ng μl^-1^ *odr-1p*::*RFP* or 5 ng μl^-1^ *myo-2p*::*RFP* co-transformation marker and 45–80 ng μl^-1^ of pBluescript plasmid DNA using standard microinjection into the distal gonad arms of young adult hermaphrodites [17]. The PRKL-1 overexpression transgene, *zyIs10*, was generated by genomic integration of the unc-4p::PRKL-1 extrachromosomal array described in [11].

### FTase Modeling

*C*. *elegans* FNTB-1 mutations were mapped to the corresponding residues of human FTase (PDB ID: 1S63) [18]. FTase rotamers were generated in PyMOL (PyMOLMolecular Graphics System, version 1.5; Schrödinger, LLC) using a backbone-dependent rotamer library.

### VC4 and VC5 morphology and PRKL-1 localization

VC4 and VC5 morphology was visualized in young adult hermaphrodites using the *cyIs4 [Pcat-1*::*GFP]* reporter. Worms were immobilized with 10 mM levamisole (Sigma) and imaged using an AxioplanII fluorescence microscope. An ectopic VC neurite was defined as a protrusion from the cell body that was greater than the length of one VC cell body (~5 μm). The relevance of the CAAX motif for plasma membrane localization in VC4 and VC5 neurons was assessed by quantifying the localization of full length and CAAX-deleted GFP::PRKL-1 (PRKL-1::ΔCTVS) in *wt* and a *vang-1(tm1422)* null backgrounds. The *unc-4* promoter was used to express GFP::PRKL-1 in VC neurons. VC4 and VC5 were scored during early L4 and identified by unc-4p::GFP::PRKL-1 fluorescence and their stereotypical positions flanking the vulva. Plasma membrane localization was quantified by binning observations into two primary categories: (1) 10 or more membrane punctae (many puncta) and (2) fewer than 10 membrane punctae (few puncta).

## Results and Discussion

### A forward genetic screen identifies five genes required to block VC neurite outgrowth along the AP axis

**Fig 1:**
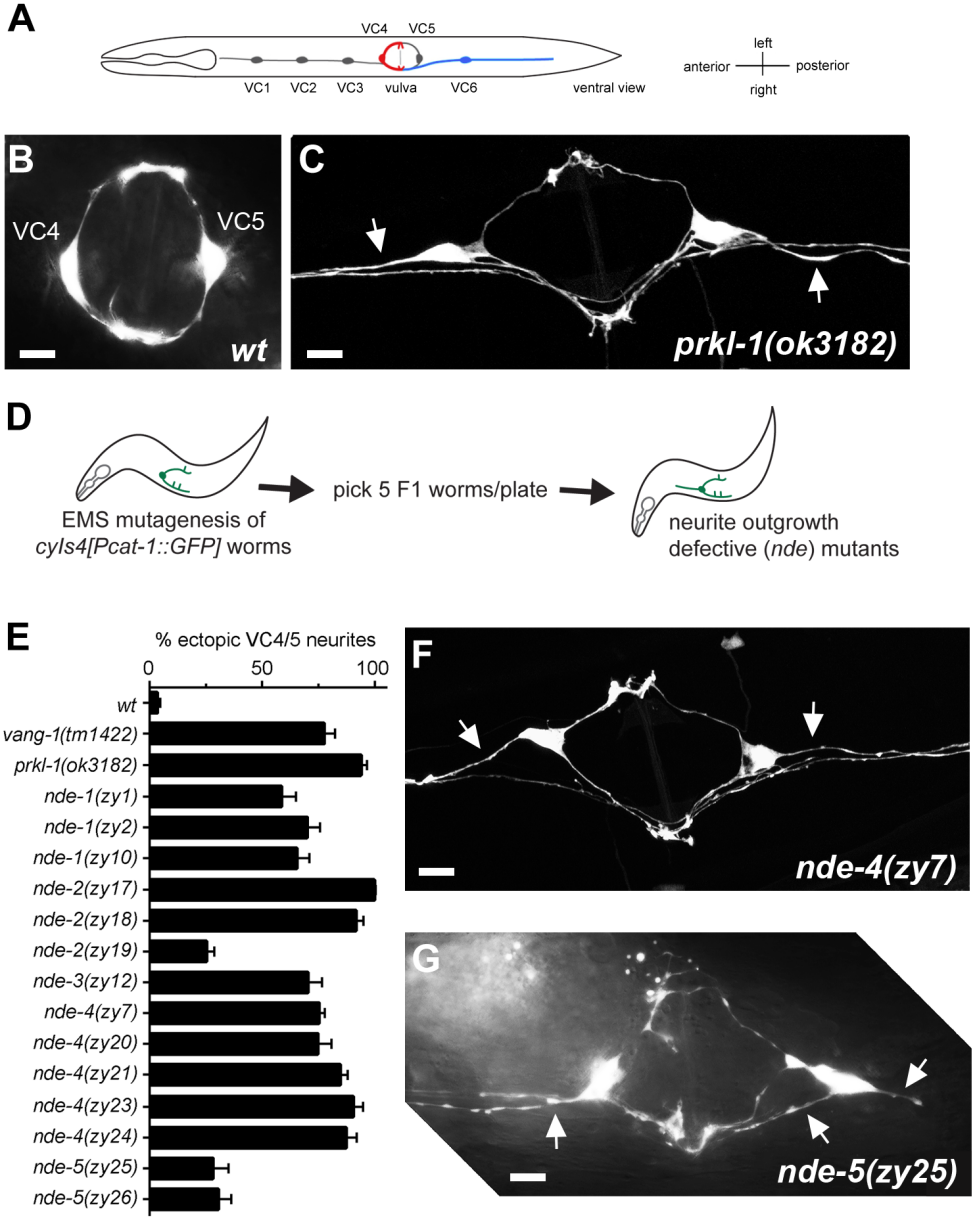
A genetic screen for VC4 and VC5 neurite outgrowth defective (Nde) mutants. (A) Worm schematic showing the position of VC1–6 (ventral view). VC4 and VC5 neurons extend neurites along a LR axis and VC1–3 and VC6 extend neurites along the AP body axis. (A) Wild-type VC4 and VC5. (B) In *prkl-1* and *vang-1* (not shown) mutants, VC4 and VC5 display ectopic neurites (arrows) directed along the AP axis. (D) Schematic outline of a forward genetic screen for VC4 and VC5 neurite outgrowth defective (*nde*) mutants. (E) Quantification of ectopic neurite defects in *nde* mutants. Graph shows the percentage of VC4 and VC5 neurons (pooled) displaying at least one ectopic neurite. Error bars represent the 95% confidence interval of the proportion. For each genotype, n>160. (F and G) Representative images of VC4 and VC5 in *nde-4* (F) and *nde-5* (G) mutants. Ectopic neurites are marked with arrows. All lines contain the *cyIs4[Pcat-1*::*GFP]* reporter background. All images are ventral views. Scale bars, 10μm.

Egg-laying in *C*. *elegans* is mediated by a circuit consisting of the VC and HSN motor neurons and the vulval sex muscles [19]. The VC neurons are a set of six motor neurons positioned along the AP axis with VC4 and VC5 flanking the vulva (Fig 1A). These neurons display stereotypical differences in axon extension along either an AP or left-right (LR) trajectory during formation of the ‘egg-laying’ circuit [20] and thus provide a morphologically simple and genetically accessible model to investigate genetic programs involved in neurite emergence. We have previously shown that a PCP-like pathway that includes *vang-1*, *prkl-1* and *dsh-1* is required in VC4 and VC5 to maintain neuronal morphology [11]. Normally, VC4 and VC5 extend two neurites along a circular path around the vulva. In PCP mutants, VC4 and VC5 will often extend an additional neurite along the AP axis directed away from the vulva resulting in distinct tripolar-like morphologies (Fig 1B and 1C). To identify new components involved in suppressing VC neurite outgrowth, we performed a forward genetic screen for neurite outgrowth defective (*nde*) mutants (Fig 1D).

A visual screen of the F2 progeny of approximately 6000 EMS mutagenized *cyIs4[cat-1p*::*gfp]* worms identified 14 mutants in which VC4 and VC5 extended neurites inappropriately along the AP axis. Since our previous study showed that *vang-1*, *prkl-1* and *dsh-1* act to block ectopic neurite formation [11], we performed standard genetic complementation tests to determine if any of these genes were identified in our screen (data not shown). This analysis followed by sequencing revealed three new alleles each of *vang-1* and *prkl-1* (*nde-1* and *nde-2* respectively) and a single new allele of *dsh-1* (*nde-3*) (Tables 1 and 2). Sequencing did not identify a polymorphism in the *prkl-1* coding region of *nde-2(zy17)* despite *zy17* failing to complement *prkl-1(ok3182)* and mapping to a small interval on LGIV that included the *prkl-1* locus (data not shown). It is possible that *zy17* represents a mutation in *prkl-1* regulatory sequence or a complex rearrangement of the *prkl-1* locus. All *vang-1*, *prkl-1* and *dsh-1* mutants displayed highly penetrant morphology defects consistent with previous findings (Fig 1E).

**Table 1:** VC4 and VC5 neurite outgrowth defective (*nde*) mutants.

Mutant	Alleles	Gene
*nde-1*	*zy1*, *zy2*, *zy10*	*vang-1*/Van Gogh
*nde-2*	*zy17*, *zy18*, *zy19*	*prkl-1*/Prickle
*nde-3*	*zy12*	*dsh-1*/Disheveled
*nde-4*	*zy7*, *zy20*, *zy21*, *zy23*, *zy24*	*fntb-1*/ β subunit of farnesyltransferase
*nde-5*	*zy25*, *zy26*	-

**Table 2:** Sequence changes in *nde* mutants.

Gene	Allele	Mutation	Sequence Change	Effect
***nde-1/vang-1***	*zy1*	G > A	atttgca**a**GGC CAG AAG	splice acceptor
	*zy2*	G > A	TTC ACA TG**A** ATT GTC	406Wstop
	*zy10*	G > A	GGA AGA CG**a**taaatgtt	splice donor
***nde-2/prkl-1***	zy17*	-	-	-
	*zy18*	C > T	GCA CCG **T**AG CTC ATC	Q19stop
	*zy19*	G > A	tcttaca**a**GTT CAT GCC	splice acceptor
***nde-3/dsh-1***	*zy12*	C > T	GTC AAA **T**AG CAA CCA	Q64stop
***nde-4/fntb-1***	*zy7*	G > A	ATG CTT **A**AA GAG TAC	E321K
	*zy20*	G > A	TGC TAC A**A**C TTC TGG	S289N
	*zy21*	C > T	ATG GAA **T**GA GAA GGC	R305stop
	*zy23*	G > A	CAC GGA G**A**A TAC ACT	G238E
	*zy24*	G > A	TGG ATG T**A**C TAC TGG	C92Y

*zy17 and *prkl-1(ok3182)* fail to complement but sequencing of *prkl-1* exons did not reveal a *zy17* polymorphism.

The seven remaining mutants were assigned to two complementation groups, *nde-4* (5 alleles) and *nde-5* (2 alleles) (Table 1). These mutants were mapped using single nucleotide polymorphisms (SNPs) between Hawaiian and Bristol (N2) strains [21] to chromosomes V and I respectively (S2 Table). The penetrance of ectopic VC neurites in *nde-4* mutants were similar in severity to those of *prkl-1* mutants (Fig 1E). In *prkl-1(ok3182)* null mutants, 94% of VC4 and VC5 neurons displayed ectopic neurites compared to 90% in *nde-4(zy23)*. *nde-5* mutants displayed less severe defects (approximately 30% ectopic neurites) compared to the others (Fig 1E). Most mutants showed qualitatively similar VC4 and VC5 morphologies (Fig 1C and 1F) except for *nde-5* mutants which often displayed multiple neurites extending from cell somas (Fig 1G). *nde-4* mutants also displayed low brood sizes, egg retention and small body size. The recovery of multiple alleles for most complementation groups suggests that the screen may have approached saturation for viable mutants.

### A farnesyltransferase is involved in VC neurite formation

**Fig 2:**
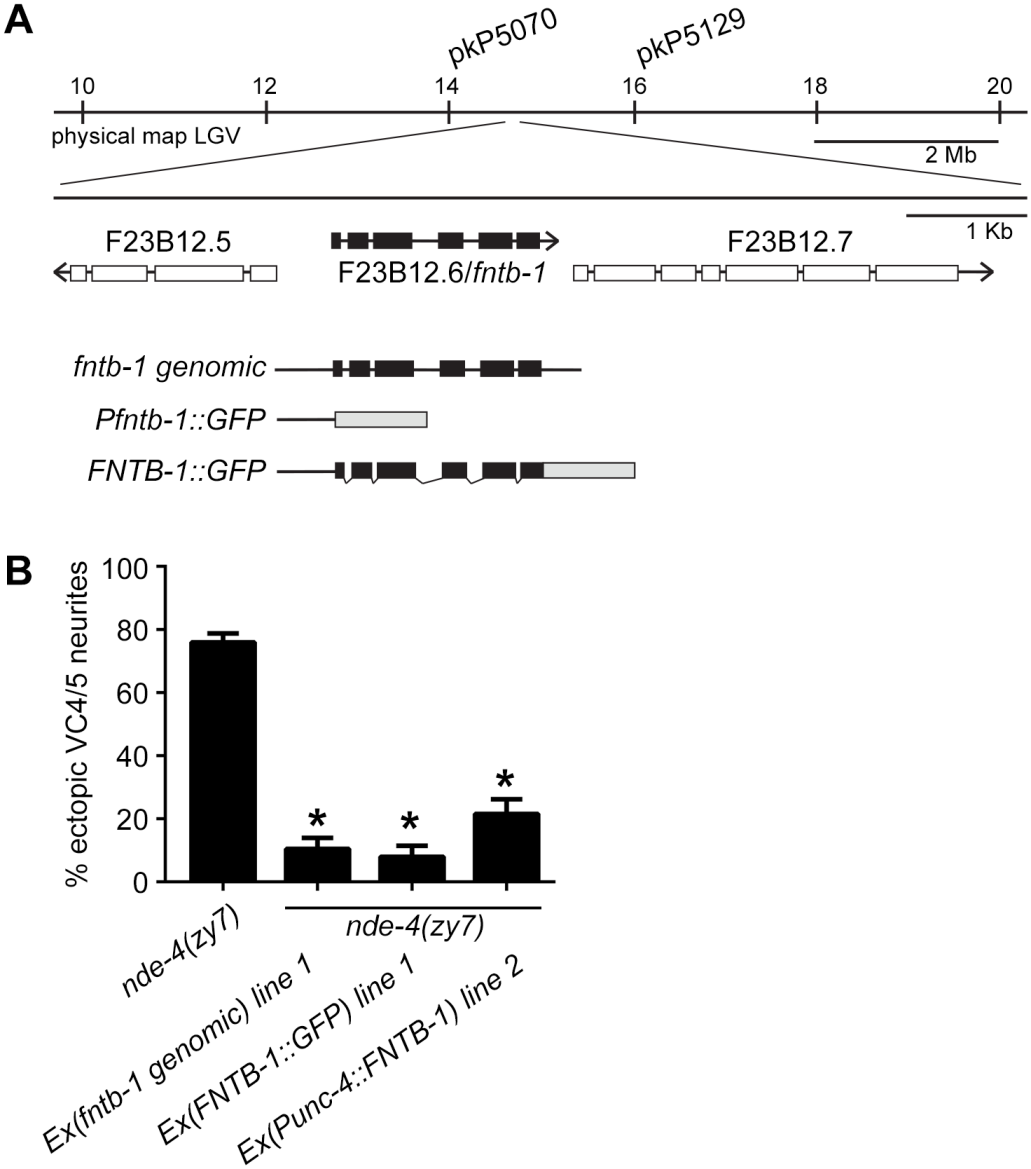
The *nde-4* gene encodes the farnesyltransferase (FTase) β subunit (*fntb-1*). (A) *nde-4* mutants were mapped to a small interval on chromosome V and rescued with a genomic fragment containing the *fntb-1* (F23B12.6) gene and an *FNTB-1*::*GFP*-translational fusion driven from the *fntb-1* promoter. Promoter and genomic regions used in rescuing constructs and a *Pfntb-1*::*GFP* transcriptional reporter are delineated. (B) Quantification of VC4 and VC5 ectopic neurite rescue in *nde-4(zy7)* mutants by *fntb-1* genomic, *FNTB-1*::*GFP* and *unc-4* promoter driven FNTB-1 constructs. Representative extrachromosomal lines are shown. Error bars represent the standard error of the proportion, n>200. *p<0.001, t test.

Because *nde-4* mutants displayed highly penetrant morphology defects and represented the largest complementation group with 5 alleles, we sought to identify the gene product to gain further insight into how VC neurite extension is regulated. While outcrossing we found that *nde-4* mutations could not be separated from our *cyIs4* reporter indicating a linkage to chromosome V. We subsequently mapped *nde-4* to a roughly 2 Mb region on chromosome V between SNPs pkP5070 (AH10) and pkP5129 (F57G8) (S2 Table). A systematic search of genes within this region using the genome browser at Wormbase.org (version WS249) revealed *fntb-1*(F23B12.6), the β subunit of farnesyltransferase (FTase), as a possible candidate (Fig 2A). A genome-wide analysis of trans-spliced mRNAs identified *fntb-1* as the upstream gene in an operon with F23B12.7 [22]. *fntb-1* was flagged as a good candidate for *nde-4* as PRKL-1 contains a carboxy-terminal prenylation signal (CAAX motif) and Prickle proteins are known to be post-translationally modified by FTases [13,14]. We confirmed *fntb-1* as the *nde-4* locus by showing that a 2.5 kb *fntb-1* genomic fragment containing 485 bp upstream of the ATG start and 332 bp downstream of the stop codon rescued *nde-4(zy7)* VC defects (Fig 2B) and by identifying single nucleotide changes in all five alleles (Table 2). *nde-4* will henceforth be referred to as *fntb-1*.

### FNTB-1 mutants are predicted to disrupt FTase catalytic activity

FTases transfer a 15-carbon prenyl (farnesyl) group from farnesyl diphosphate (FPP) to CAAX-motif containing proteins. They function as heterodimers consisting of a regulatory α subunit (FNTA) and catalytic β subunit (FNTB) that contains the substrate and FPP-binding pockets [12]. FNTA-1 and FNTB-1 are the sole worm orthologues of the alpha and beta-subunits respectively. In this study, we will focus primarily on the beta-subunit. Compared to human FNTB, *C*. *elegans* FNTB-1 shows strong sequence conservation (46% amino acid identity, 60% amino acid similarity) across its entire length (S1 Fig).

**Fig 3:**
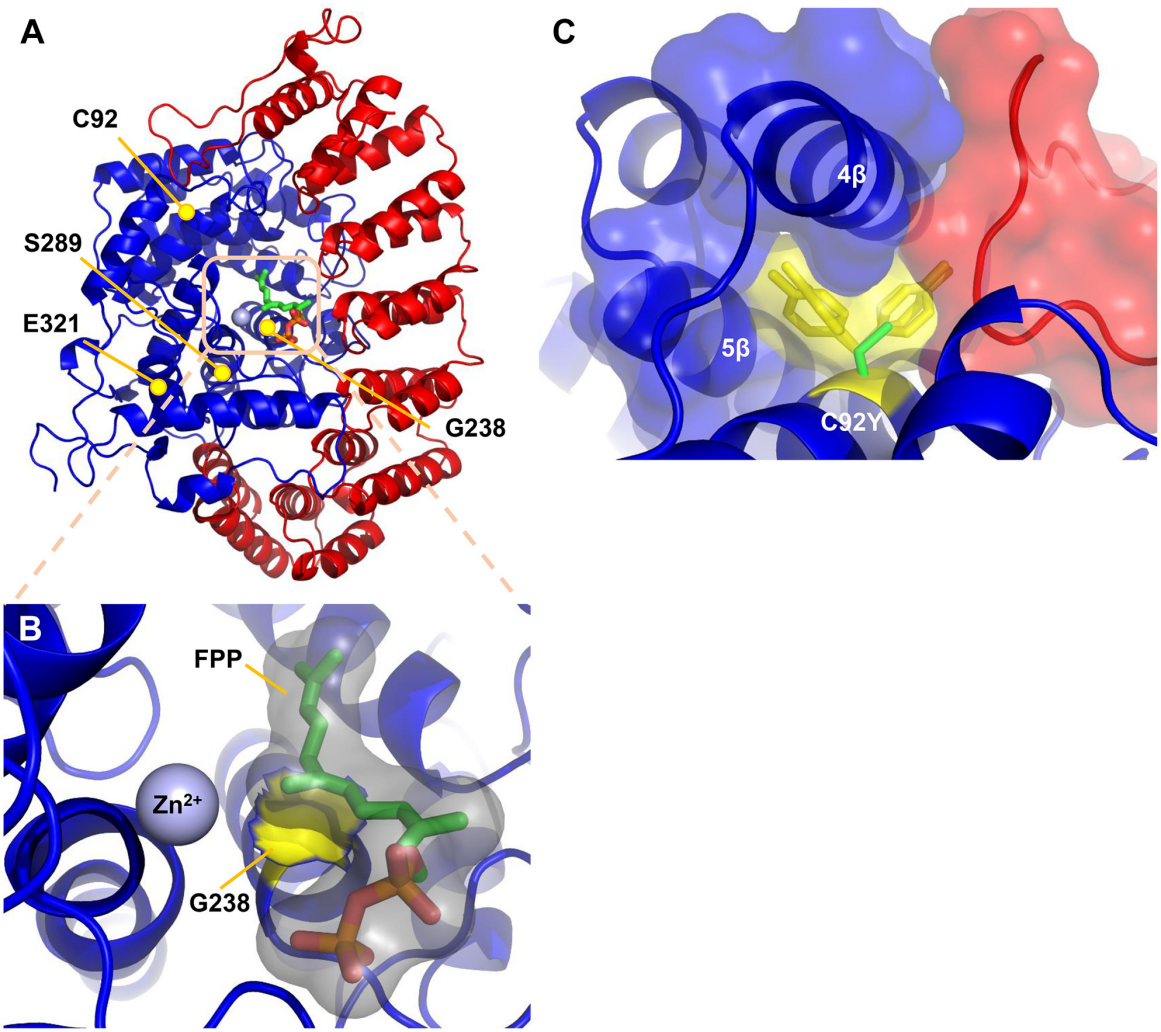
FNTB-1 missense mutations mapped to human FTase. (A) Crystal structure of the human protein FTase (PDB ID: 1S63) heterodimer (α subunit; red and β subunit; blue). Yellow circles mark the location of residues C104, G250, S301 and Q336 of the worm FTase. Lipid substrate farnesyl diphosphate, (FPP: isoprenoid moiety, green; diphosphate, red) and zinc ion (grey sphere) mark the site of catalysis. (B) Enlarged view of the FTase active site. G250 (yellow surface representation) makes van der Waals contact with FPP (grey surface). Any mutation at position 250 would result in a larger side chain that would affect FPP binding. (C) Enlarged view around residue C92 in worm FTase (C104 human FTase; green stick). The four major rotamers for the C104Y mutation and their corresponding van der Waals surfaces are shown in yellow. A van der Waals surface calculated around the alpha subunit (red) and beta subunit (blue) is shown. Any tyrosine rotamer results in a steric class with the protein. This mutation is predicted to compromise the packing and the stability of the enzyme.

Of the five *fntb-1* mutants identified in our screen, four are missense mutations (*zy7*, *zy20*, *zy23*, *zy24*) that alter conserved residues and one is a nonsense mutant (*zy21*) that introduces a stop codon at arginine 305 (S1 Fig). Interestingly, *zy21* animals are viable despite the existence of a deletion allele, *fntb-1(ok590)*, generated by the *C*. *elegans* Gene Knockout Consortium that is not viable. To understand how *fntb-1* mutations might disrupt protein function we used the crystal structure of human FTase (PDB ID: 1S63) [18] to map the location of mutations in the highly homologous worm FTase (Fig 3A).

This analysis suggests that the FNTB-1 truncated protein generated in *zy21* would lack residues and secondary structure that form the FPP-binding catalytic domain and thus should completely lack enzymatic activity. How to explain the non-viability of *ok589*? A likely explanation is that because the *ok590* deletion removes approximately 100bp upstream of the *fntb-1/F23B12*.*7* operon start in addition to *fntb-1* coding region, it might also disrupt the expression of the co-transcribed F23B12.7 gene. The non-viability of *ok590* may thus result from loss of F23B12.7 singly or in combination with *fntb-1*. In contrast to *fntb-1* mutants, *ok269*, a deletion in *fnta-1*, is non-viable. FNTA is a shared subunit in both FTase and another prenyltransferase, geranylgeranyltransferase (GGTase), and may therefore participate in the post-translational modification of a greater complement of substrates, some of which may be essential for viability [12].

How might the other conserved residues disrupted in our *fntb-1* mutants affect FTase function? The G238E substitution in *zy23* maps to G250 in human FNTB. This residue is associated with the active site. Any substitution at position G238 would result in an elongation of the side chain and therefore interfere with the productive binding of the FPP lipid substrate (Fig 3B). *zy24* (C92Y) maps to human FNTB residue C104. A tyrosine substitution at this position is predicted to compromise the packing and stability of the enzyme as any tyrosine rotomer would result in a steric clash with either the alpha-helices of FNTB or a loop of FNTA (Fig 3C). VC morphology defects in *zy23* and *zy24* are quantitatively similar to the *zy21* null, suggesting that they may also completely disrupt protein function. *zy7* (E321K) and *zy20* (S289N) substitutions map to residues Q336 and S301 in human FNTB. The consequences for substitutions at these residues are less clear but they may also compromise protein stability by disrupting alpha helices. It is important to note however that in the absence of empirical data, this analysis is speculative.

### FNTB-1 acts in VC4 and VC5

To determine where *fntb-1* is expressed during the period of VC neurite outgrowth we made GFP promoter and protein fusions. We showed that a genomic fragment containing 485 bp of *fntb-1* promoter sequence was able to rescue VC morphology defects in *fntb-1(-/-)* mutants. This promoter was also able to rescue VC defects when used to drive *fntb-1* cDNA expression (Fig 2A and 2B) suggesting that the regulatory elements contained therein are sufficient to properly express *fntb-1* and therefore suitable for GFP reporters.

**Fig 4:**
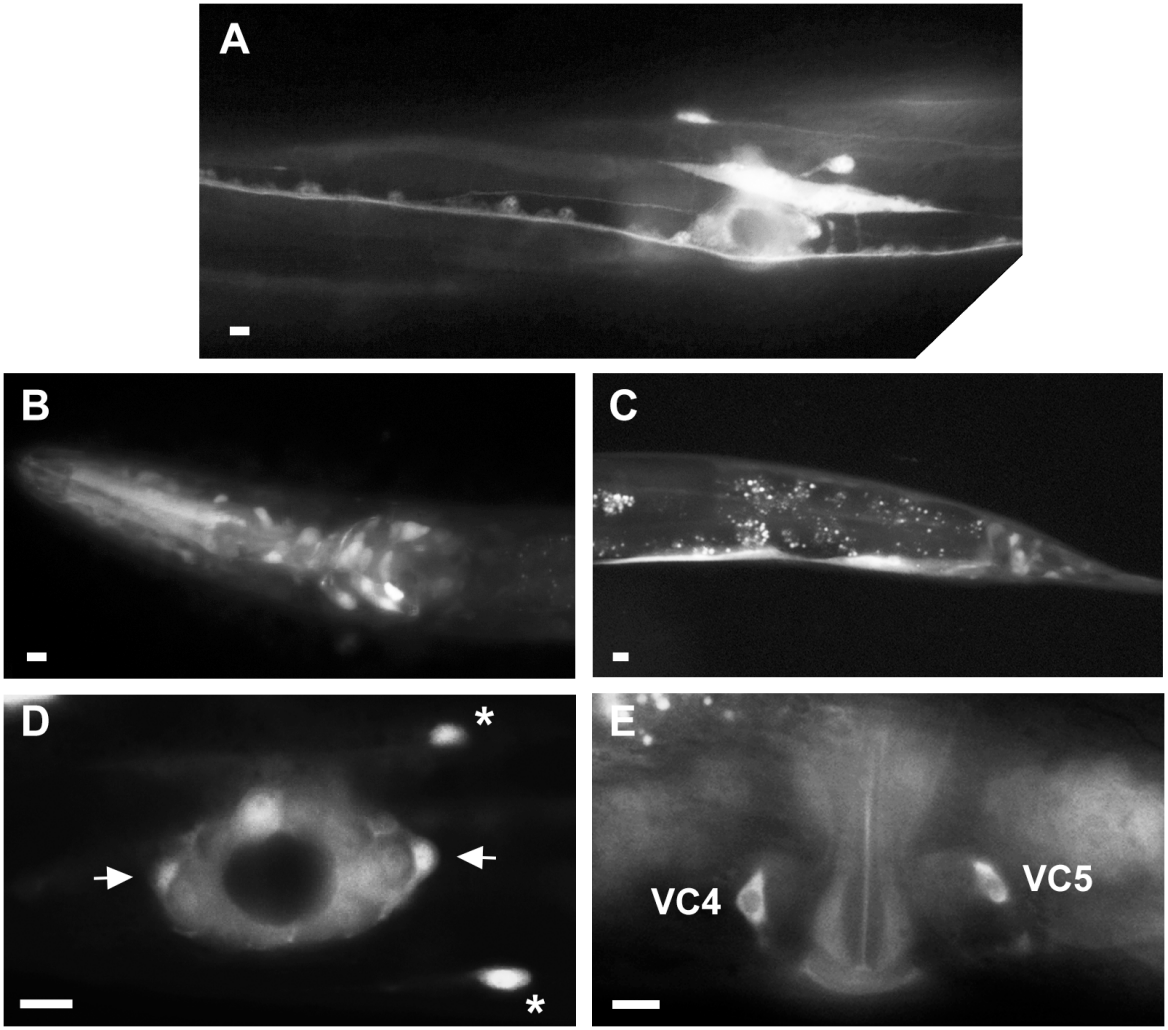
*fntb-1* is expressed in VC4 and VC5 neurons. (A) *Pfntb-1*::*GFP* expression in the mid-body region of an L4 hermaphrodite. *Pfntb-1*::*GFP* expression in the head (B) and tail (C) region of an L4 hermaphrodite. The *fntb-1* promoter is active in neurons, vulval and rectal epithelial cells and in muscle cells. (D) *Pfntb-1*::*GFP* transcriptional activity in VC4 and VC5 (arrows), HSNs (asterisks) and vulval epithelial cells at the mid-L4 stage vulval region. (E) Adult vulval region showing FNTB-1::GFP protein fusion predominantly localized to the cytoplasm of VC4 and VC5 neurons. Panels A, D and E show ventral views. Panels B and C show side views. Scale bars, 10μm.

In L4 animals when VC neurite outgrowth is taking place, *fntb-1* promoter activity was found in VC neurons and vulval cells (Fig 4A and 4D). This expression pattern is similar to that of *prkl-1* at the same stage [11]. We previously showed that *prkl-1* acts cell autonomously to block ectopic VC neurite outgrowth [11]. We therefore asked if *fntb-1* was required in VC neurons as would be expected if PRKL-1 was a target of FTase activity. The *unc-4* promoter was used to drive *fntb-1* expression in a subset of neurons including the VC neurons. We found that *unc-4p*::*FNTB-1* expression was able to restore proper VC morphology in *fntb-1(-/-)* mutants indicating that *fntb-1* acts cell autonomously to promote proper VC morphology (Fig 2B). In addition to VC neurons, *fntb-1* was expressed in motor neurons in the ventral nerve cord, mid-body region neurons (HSNs and CANs), and various neurons in the head and tail (Fig 4A–4C). In non-neural tissue, *fntb-1* expression was observed in body wall and pharyngeal muscle and rectal epithelial cells (Fig 4A–4C).

To determine where FNTB-1 accumulates in VC neurons, we generated transgenic animals carrying an *fntb-1* cDNA fused in frame to GFP and driven from the *fntb-1* promoter. Expression from the *Pfntb-1*::*FNTB-1*::*GFP* transgene was able to rescue *fntb-1* VC defects (Fig 2B) suggesting that the C-terminal GFP tag does not interfere with FNTB-1 function. In VC neurons, FNTB-1::GFP protein accumulated in the cell soma and along axons but was excluded from the nucleus as expected for a cytoplasmic protein (Fig 4E).

### Farnesylation is important for PRKL-1-mediated inhibition of ectopic neurites in VC neurons

**Fig 5:**
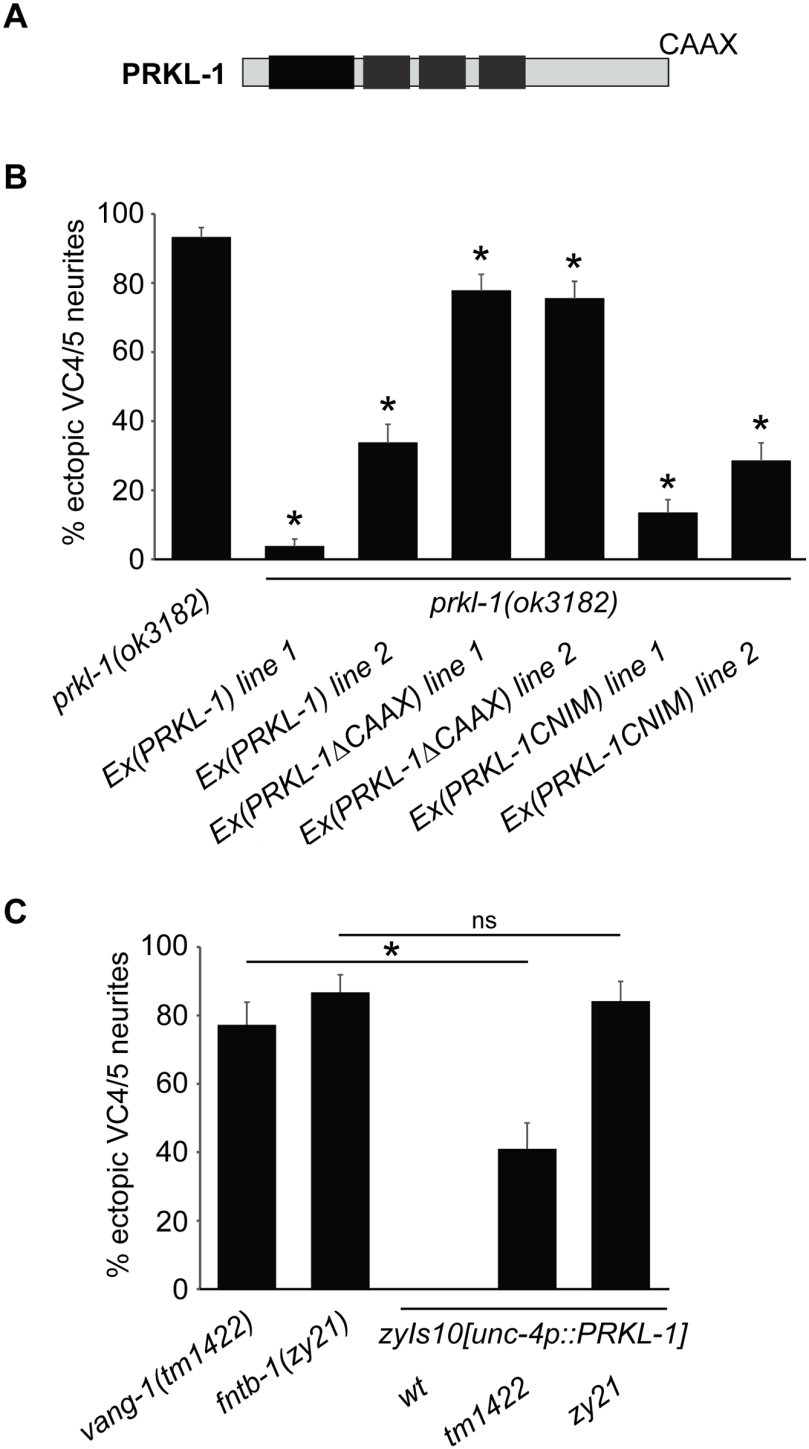
The PRKL-1 CAAX motif is necessary for normal VC4 and VC5 morphology. (A) Schematic of PRKL-1 showing conserved domains (black boxes) and C-terminal CAAX motif. (B) Expression of CAAX deleted PRKL-1 does not rescue VC4 and VC5 morphology defects in *prkl-1* mutants as well as full length PRKL-1. Replacement of the PRKL-1 CAAX motif with the CNIM motif from MIG-2 resembles wildtype PRKL-1 in rescuing VC4 and VC5 morphology defects. (C) PRKL-1 overexpression can rescue VC morphology defects in *vang-1* mutants but not *fntb-1* mutants. All transgenic lines in B and C contain *unc-4* promoter-driven PRKL-1 constructsand all lines contain the *cyIs4[Pcat-1*::*GFP]* reporter background. Error bars represent the 95% confidence interval of the proportion, n>150. *p<0.001, t test.

A farnesyl group is covalently attached to C-terminal CAAX motif containing substrates (Fig 5A). Prickle orthologues with mutated or deleted CAAX motifs are not farnesylated [13,14]. To determine the functional importance of the PRKL-1 CAAX motif, CTVS, we compared the ability of *unc-4* promoter-driven full length PRKL-1 and PRKL-1::ΔCTVS to restore proper VC morphology in *prkl-1* mutants. We found that PRKL-1::ΔCTVS expression was far less effective at rescuing ectopic VC neurites in *prkl-1* mutants compared to full length PRKL-1 (Fig 5B) indicating that the CAAX signal is important. The mild but significant decrease in ectopic neurites when PRKL-1::ΔCTVS was expressed in *prkl-1(-/-)* mutants may be the result of overexpression from the multi-copy transgenic array. Alternatively, functional non-farnesylated PRKL-1 may normally play some role in blocking ectopic neurite formation.

We next sought to determine the effect of replacing the PRKL-1 CAAX motif with a heterologous prenylation signal. We reasoned that if the PRKL-1 CTVS motif acts as a prenylation signal, then substitution with a CAAX motif from a heterologous protein should not interfere with PRKL-1 function. The Ras and Rho family of small GTPases are well characterized prenylation substrates for FTases [23]. We therefore substituted the C-terminal 15 amino acids of PRKL (VRMAKKKKSSRCTVS) with the corresponding region (LHPKPQKKKKSCNIM) from MIG-2, a *C*. *elegans* Rho GTPase [24]. We swapped an extended C-terminal region as CAAX motifs are usually preceded by a poly-basic region that is important for prenylation [25]. We found that expression of PRKL-1::CNIM is largely indistinguishable from full length PRKL-1 in rescuing *prkl-1* VC morphology defects (Fig 5B). Combined, CAAX deletion and substitution support the notion that the CAAX motif is important for PRKL-1 to maintain normal VC morphology.

Previous findings showed that PRKL-1 overexpression is able to partially rescue the ectopic VC neurite defects in *vang-1* mutants suggesting that PRKL-1 acts downstream of VANG-1 [11]. We therefore asked if PRKL-1 overexpression could also rescue VC defects in *fntb-1* mutants. A positive outcome would suggest that prenylation is not a necessary requirement for PRKL-1 to block ectopic neurite outgrowth. We found that PRKL-1 overexpression does not rescue *fntb-1* mutants (Fig 5C) consistent with the notion that PRKL-1 prenylation is important for maintaining VC morphology. However, we cannot exclude the possibility that FTase modifies another component, in addition to PRKL-1, that is also important for blocking ectopic VC neurite formation.

### Farnesylation is important for PRKL-1 membrane localization

Asymmetric Prickle accumulation on the plasma membrane of *Drosophila* epithelial cells [26] and vertebrate cells during convergent extension [27,28] is thought to play a key role in PCP signaling. In *Drosophila*, the requirement for Prickle prenylation in mediating membrane localization appears to be both prickle isoform and cell context-dependent [14,29,30]. In zebrafish, Prickle membrane localization in cells undergoing gastrulation is partially dependent on its CAAX motif [31]. Prenylation is also important for activity that does not reside at the plasma membrane. Prenylation-dependent nuclear localization of Prickle has been shown to promote neuronal migration [32] and polarity establishment in early embryos [33].

**Fig 6:**
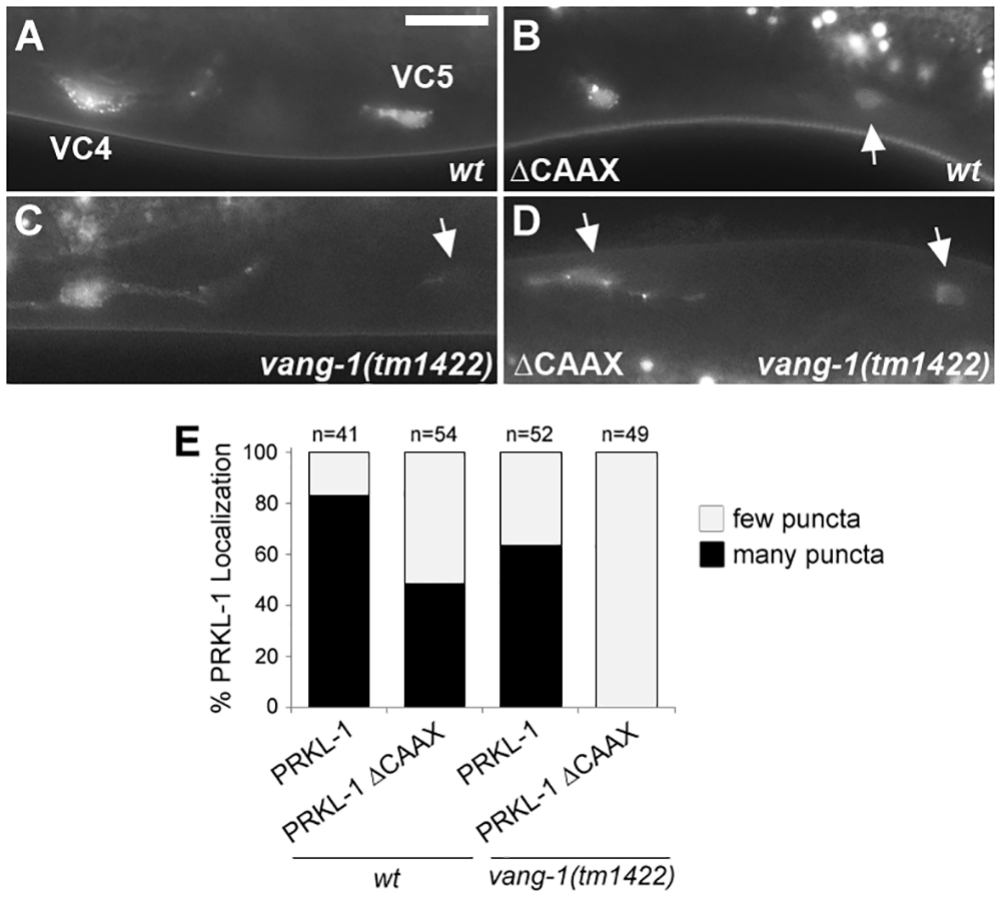
The PRKL-1 CAAX motif is important for membrane localization. (A) Transgene expression of a GFP::PRKL-1 fusion shows punctate localization on the plasma membrane of VC4 and VC5 (many puncta) at the early L4 stage. Representative images showing the localization of a CAAX-deleted PRKL-1::GFP fusion in a *wild-type* (*wt*) background (B) and full length GFP::PRKL-1 in a *vang-1* mutant background (C). In both panels B and C, a localization pattern resembling full length PRKL-1 (many puncta) or diminished membrane localization (few or no puncta, arrows) are observed. (D) Expression of a CAAX-deleted PRKL-1 construct in a *vang-1* mutant background shows loss of plasma membrane localization in VC4 and VC5 (few/no puncta). (E) Quantification of full length GFP::PRKL-1 and GFP::PRKL-1 ΔCAAX membrane distribution in *wt* and *vang-1* mutants in early L4 stage VC4 and VC5.

VC4 and VC5 extend neurites around the vulval epithelium beginning at the early L4 stage. During this period, PRKL-1 was found in the cytoplasm and in a punctate, but not an obvious asymmetric, distribution on the plasma membrane of VC neurons [11]. Since PRKL-1::ΔCAAX expression is unable to fully restore proper VC morphology in *prkl-1* mutants, we asked if the CAAX domain is required for PRKL-1 localization to the plasma membrane. Because our *fntb-1* mutants and the VC4/VC5 *cyIs4[cat-1p*::*GFP]* reporter transgene used in the genetic screen (Fig 1) are tightly linked on LGV we were unable to assess GFP::PRKL-1 localization directly in *fntb-1* mutants. We therefore compared the localization of GFP::PRKL-1 and GFP::PRKL-1::ΔCTVS in VC4 and VC5 using the *unc-4* promoter-driven transgenes scored in Fig 5 for *prkl-1* rescue (Fig 6A and 6B). Since PRKL-1 and PRKL-1::ΔCTVS displayed significantly different abilities to rescue VC4 and VC5 morphology defects, we expected to observe differences in PRKL-1 subcellular localization. We found that the proportion of VC4 and VC5 neurons showing many membrane puncta in worms expressing GFP::PRKL-1::ΔCTVS (48%, n = 54) was less than GFP::PRKL-1 expressing worms (83%, n = 41) (Fig 6F). These results suggest that the CAAX domain, and therefore farnesylation, is important for PRKL-1 localization to the membrane of VC neurons.

We next assessed the involvement of VANG-1, the worm orthologue of *Van Gogh*, in PRKL-1 and PRKL-1::ΔCTVS localization. In *Drosophila* epithelial cells, *Van Gogh* recruits *Prickle* to the plasma membrane to mediate planar cell polarity [29,34]. Consistent with previous findings [11], PRKL-1::GFP localization, as defined by the proportion of neurons with many small membrane punctae, did not appear to differ greatly in *vang-1* mutants (64%, n = 52) compared to wild-type (83%, n = 41) (Fig 6C and 6F). In contrast, PRKL-1::ΔCTVS membrane localization showed a striking decrease when expressed in *vang-1* mutants (0%, n = 49) (Fig 6D and 6F). Combined, these findings are consistent with the notion that PRKL-1 recruitment to the VC plasma membrane is important for blocking AP-directed neurite formation and that VANG-1 and FTase act independently, or at least partially, in PRKL-1 membrane localization.

However, if membrane localization is sufficient for proper neurite formation, then PRKL-1::ΔCTVS localization to the membrane of 48% of neurons (n = 54) in wild type animals appears at odds with the almost complete failure of PRKL-1::ΔCTVS to rescue *prkl-1(-/-)* neurite defects (Fig 5B) and the high penetrance of defects in *fntb-1(-/-)* mutants. These observations suggest that a C-terminal farnesyl group may be involved in more than simply mediating membrane insertion. One possibility is that PRKL-1 farnesylation acts to stabilize or promote an interaction between VANG-1 or another pathway component required for neurite inhibition. Such a notion has been proposed in *Drosophila*, where *Prickle* farnesylation is believed to promote physical association with Van Gogh to organize the polarity of wing cells [14].

## Conclusions

We show for the first time that an FTase acts to establish proper neuronal morphology by blocking supernumerary neurite formation in *C*. *elegans*. The results of our genetic screen are consistent with previous work showing that a PCP-like pathway consisting of *prkl-1*, *vang-1* and *dsh-1* acts to establish and maintain neuronal soma morphology. We show that localization of PRKL-1, an FTase substrate, to the membrane of neuronal somas is partially dependent on both its CAAX motif and recruitment by VANG-1. During establishment of epithelial planar polarity in *Drosophila*, *Prickle* farnesylation has been shown to be important in some cellular contexts but not others. *Prickle* farnesylation has also been linked to both membrane and nuclear targeting in PCP-mediated processes as varied as epithelial cell polarity and neuronal and mesenchymal migration. These findings combined with our own highlight the diversity of roles played by farnesylation in the context of PCP-like signaling.

## Supporting Information

S1 Fig The *C*. *elegans* FTase β subunit (FNTB-1) is highly conserved. An alignment of the *C*. *elegans* and human FTase-beta subunits. The position and identity of molecular lesions in *fntb-1* are indicated. *Ce*, *C*. *elegans* (GenBank accession number CAB01167); *Hs*, human (GenBank accession number NP002019). ClustalW alignment (version 6.0).
(TIF)

S1 Table Primer sequences for molecular cloning. (PDF)

S2 Table SNP mapping of *nde-4* and *nde-5*. (PDF)
