# Supplementary figures and images for: A Farnesyltransferase Acts to Inhibit Ectopic Neurite Formation in C. elegans
Source: PLoS One. 2016 Jun 14;11(6):e0157537. doi: 10.1371/journal.pone.0157537 (PMC4907426; doi:10.1371/journal.pone.0157537)

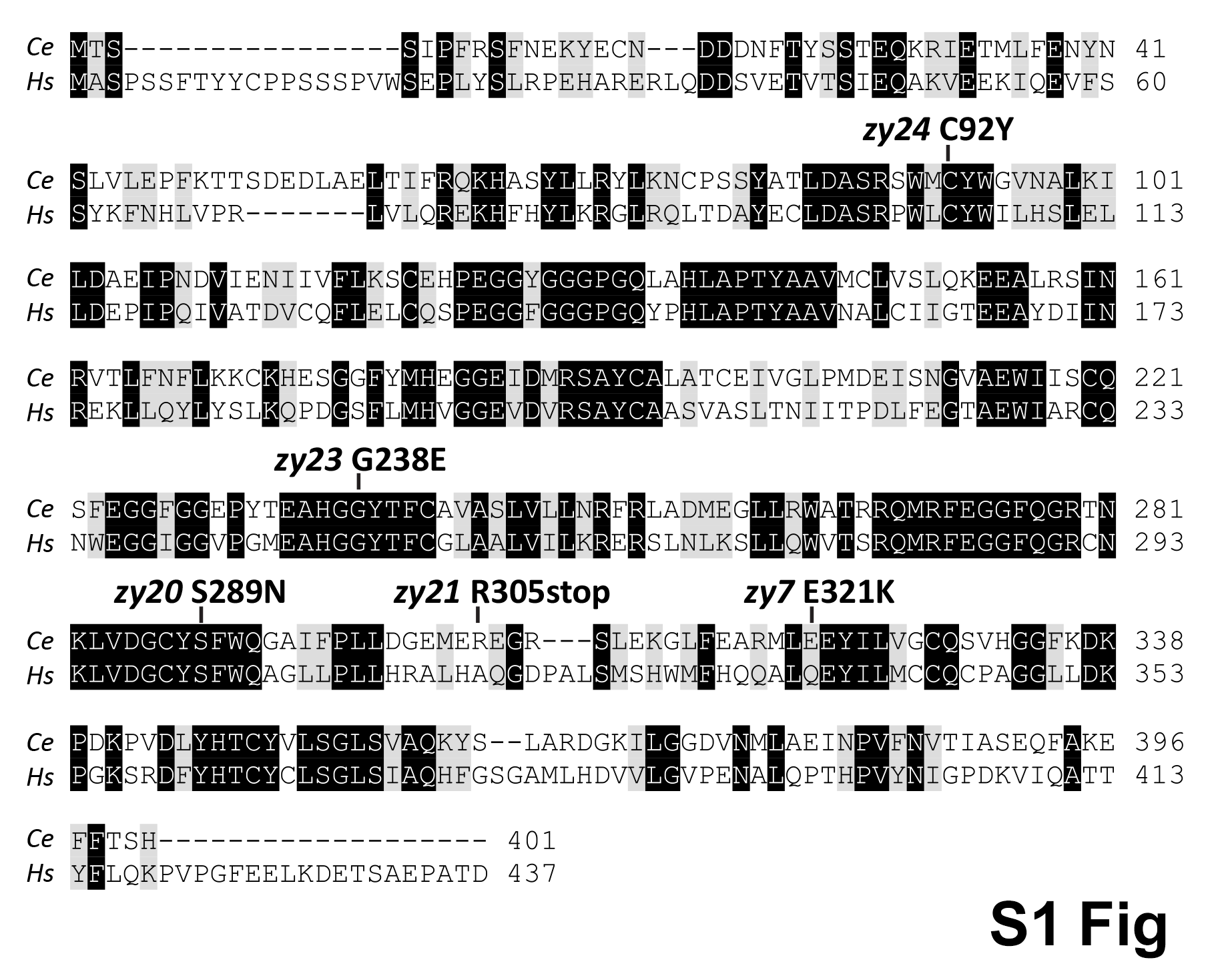

Supplement: S1 Fig — An alignment of the C. elegans and human FTase-beta subunits. The position and identity of molecular lesions in fntb-1 are indicated. Ce, C. elegans (GenBank accession number CAB01167); Hs, human (GenBank accession number NP002019). ClustalW alignment (version 6.0). (TIF) [file pone.0157537.s001.tif]
