# Supplementary material for: A Farnesyltransferase Acts to Inhibit Ectopic Neurite Formation in C. elegans
Source: PLoS One. 2016 Jun 14;11(6):e0157537. doi: 10.1371/journal.pone.0157537 (PMC4907426; doi:10.1371/journal.pone.0157537)
Supplement: S1 Table — (PDF) [file pone.0157537.s002.pdf]

Supplemental Table 1. Primer sequences for molecular cloning.

Construct	Primer Sequence (5'-3')
fntb-1 genomic fragment	F: TCGTTCTCAACGGCACCGGGAA
	R: TCCGTTGGCTGCTTCGGCTTTG
FNTB-1cDNA::GFP	F: ATGACATCTTCGATCCCATTC
	R: ATGAGAAGTGAAGAACTCCTTG
fntb-1p::GFP	F: TCGTTCTCAACGGCACCGGGAA
	R: ATGGGATCGAAGATGTCATCTC
unc-4p::FNTB-1::GFP	F: TCACTCACAACGATGGATACG
	R: ACGGAATGGGATCGAAGATGTCATTTTCACTTTTTGGAAGAAGAAGATCC
	F: ATGACATCTTCGATCCCATTCCGT
	R: CGTACGGCCGACTAGTAGGAAACAGTTATG
	F: CCACCTCTGTCTTCAAGGCG
	R: GGTATATTGGGAATGTATTCTGTC
unc-4p::GFP::PRKL-1ΔCTVS	F: ATGAGCGAACGAATTCGCCGTC
	R: TCATCTGGAACTTTTCTTTTTCTTTGCCATTCG
unc-4p::GFP::PRKL-1CNIM	F: TCTTGTTGAATTAGATGGTGATG
	R: AGACTTCTTCTTTTTCTGTGGTTTCGGATGAAGACCGCCTCCCGACATCATC
	F: AAACCACAGAAAAAGAAGAAGTCTTGCAATATTATGTGAGGATCCCCGGGATTG
	R: CGTACGGCCGACTAGTAGGAAACAGTTATG
	F: TGAAGGTGATGCAACATACG
	R: TTTGACACCAGACAAGTTGGT
