# Supplementary material for: A Farnesyltransferase Acts to Inhibit Ectopic Neurite Formation in C. elegans
Source: PLoS One. 2016 Jun 14;11(6):e0157537. doi: 10.1371/journal.pone.0157537 (PMC4907426; doi:10.1371/journal.pone.0157537)
Supplement: S2 Table — (PDF) [file pone.0157537.s003.pdf]

Supplemental Table 2. SNP mapping of *nde-4* and *nde-5*.

**LGI**	**Y54E10A**	**E01A2**	**F57C9**	**C30F8**	**C06A5**	**K04F10**
**physical**	**3.17**	**4.1**	**4.85**	**5.1**	**5.9**	**6.3**
**genetic**	**-4.5**	**-1.69**	**-0.7**	**-0.4**	**0.64**	**0.91**
detection of SNP from 131 *nde-5* homozygous mutants	yes	yes	no	yes	yes	yes
**LGV**	**R11D1**	**AH10**	**R02D5**	**T09F5**	**F57G8**	**Y51A2A**
**physical**	**12.7**	**14.1**	**14.5**	**15.1**	**16.3**	**18.5**
**genetic**	**4.57**	**6.1**	**6.5**	**7.5**	**10**	**17**
detection of SNP from 109 *nde-4* homozygous mutants	yes	yes	no	no	yes	yes

nde-4 is located on chromosome V between SNPs AH10 and F57G8. *nde-5* is located on chromosome I between SNPs E01A2 and C30F8. SNP mapping was performed according to Wicks *et. al.*, Nat. Genet. 2001 using SNP markers described therein. 109 and 131 homozygous mutant recombinants from N2/CB4856 worms were used to map *nde-4* and *nde-5* respectively.
